# gammaSTAR: A framework for the development of dynamic, real‐time capable MR sequences

**DOI:** 10.1002/mrm.30573

**Published:** 2025-05-20

**Authors:** Simon Konstandin, Matthias Günther, Daniel C. Hoinkiss

**Affiliations:** ^1^ Imaging Physics Fraunhofer Institute for Digital Medicine MEVIS Bremen Germany; ^2^ Physics/Electrical Engineering University of Bremen Bremen Germany; ^3^ mediri GmbH Heidelberg Germany

**Keywords:** magnetic resonance imaging, MR pulse sequence programming, multicenter studies, platform independent, reproducibility

## Abstract

**Purpose:**

To present the real‐time capability and advanced MR sequence library of the MR sequence development framework gammaSTAR.

**Methods:**

The presented platform consists of four different components: (1) a frontend for sequence development combined with a Python backend for sequence generation; (2) a Lua backend for the creation of hardware instructions; (3) a vendor‐specific driver for translation of these instructions into scanner‐specific objects; and (4) an interface for real‐time feedback capability. In vivo measurements of the same volunteer were performed for comparison of imaging and spectroscopy sequences implemented in this framework with those of one main vendor (Siemens Healthineers) at magnetic field strengths of 3 T and 1.5 T. Prospective motion correction was integrated into a spin echo EPI sequence to demonstrate the real‐time feedback capability.

**Results:**

The imaging and spectroscopy results of the gammaSTAR sequences show very similar image contrasts and qualities compared to those by the vendor. ADC maps were calculated and show values of (0.80 ± 0.14)10^−3^ mm^2^/s in white matter. Results of pseudo‐continuous arterial spin labeling gradient and spin‐echo (pCASL GRASE) and 3D radial UTE imaging demonstrate the ability to run complex sequences without long sequence preparation times. Prospective motion correction is possible by means of real‐time feedback and shows much fewer movement artifacts with mean voxel displacement of 1.63 mm (uncorrected) versus 0.37 mm (corrected). All images were reconstructed using the vendor's reconstruction pipeline.

**Conclusion:**

The platform gammaSTAR allows for MR sequence development with real‐time feedback capability demonstrated by a large number of MR sequences and applications.

## 1 INTRODUCTION

MRI is widely used for disease detection, diagnosis, and treatment monitoring. Its diverse options for creating different contrasts can make controlling the various hardware components extremely complex for MR sequence developers. Manufacturer‐specific development platforms are usually available for combining RF pulses, magnetic field gradients, trigger pulses, and acquisition events. These frameworks with their own definitions of standard pulses are highly vendor‐dependent, which hampers an easy and fast MR sequence exchange between research sites with different types of MR scanners, even for devices from the same manufacturer but with different software versions. A unified programming interface is desirable to circumvent this problem and to simplify and accelerate sequence implementation by creating and sharing reusable sequence modules and increasing accessibility by providing easy‐to‐learn script languages instead of low‐level C‐based code.

The impact of different scanner hardware and imaging protocols on the study outcome was previously shown in a multi‐vendor study on compartment volume precision in the Alzheimer's Disease Neuroimaging Initiative cohort.^1^ Another group demonstrates that even software upgrades have effects comparable to those of using different MR scanners at two timepoints.^2^ Therefore, multicenter studies should preferably be performed on the same manufacturer scanner type with the same software version to avoid changes between MR sequences.^3^ For instance, different vendor definitions of standard sinc RF pulses (e.g., time‐bandwidth product or filter function) result in varying slice profiles that strongly differ from the nominal slice thickness. Different gradient hardware specifications can also lead to temporal shifts in the vendors' product sequence schemes and thus to slightly different image contrasts. These reasons prevent the scientific progress and transfer of research results, which is why a vendor‐independent sequence development framework for rapid prototyping is essential for inter‐vendor reproducibility of quantitative MRI.^4^

There are already a small number of alternative sequence programming platforms.^5^, ^6^, ^7^, ^8^, ^9^ Some of them are still kept up to date and range from open‐source to commercial products (RTHawk and SpinBench, HeartVista). Recently, a journal paper was published that demonstrates a validation framework for open‐source MR sequences,^10^ which also summarizes the open‐source projects.

The most prominent sequence format Pulseq^5^ offers programming environments for MATLAB (The MathWorks) and Python.^11^, ^12^ Interpreters are currently available for running sequences on various vendor machines like Siemens, GE, Philips, and (FL)OCRA systems. Pulseq focusses on easy and fast sequence implementation with the accompanying lack of sequence real‐time interaction. The sequence export consists of static hardware events for one specific protocol parameter configuration so that dependencies and sequence logic no longer exist after conversion to the Pulseq format. The sequence must also be recompiled for MR scanners with varying hardware limitations and use of inline vendor reconstruction pipelines is limited. To the best of the authors' knowledge, currently available non‐commercial MR sequence development platforms^5^, ^6^, ^7^, ^8^, ^9^ do not offer interfaces for real‐time sequence adaptation.

In this work, the basic concepts of portable and platform‐independent MR pulse sequence programs^13^ published previously are used to present a platform for developing and executing advanced MRI sequences with real‐time capabilities, called gammaSTAR.^14^ The main focus of this new iteration of the platform is allowing real‐time execution of the MR sequences in gammaSTAR extending the scope of use to long and advanced imaging protocols and real‐time applications (e.g., frequency drift and prospective motion correction). This is done by using a suitable sequence format that still contains all sequence logic and mathematical expressions when bringing to the MR hardware, and by using the internal calculation logic that allows execution of long sequences without high memory consumption. This platform should also be user‐friendly through simple scripting in the lightweight scripting language Lua. A separate graphical user interface (GUI) for selecting sequences and setting up protocols at the MRI makes it easier to operate the framework at different types of MR scanners. The goal in the sequence development process is to achieve modularity through the combination of individual and self‐containing sequence building blocks.

The first section describes the sequence generation process within the gammaSTAR framework. The sequence format is shown alongside different frontend tools to simplify and accelerate sequence development. The following section explains how this sequence is translated to hardware events at the MR scanner, focusing on the hardware‐specific driver software. It follows an explanation of the real‐time capability, which is used by a buffered sequence execution and enables a new scope of the software. Finally, in vivo results are presented and compared to product sequences at two different magnetic field strengths. Real‐time applications show the capability of sequence adaptations during the scan.

## 2 METHODS

The gammaSTAR framework can be separated into four different components, which are described in detail below: (1) the sequence development user interface and Python backend, which are used to create gammaSTAR sequence files; (2) the Lua backend to interpret the sequence files and to create hardware instructions; (3) the scanner‐specific driver software for translating the instructions into scanner‐specific commands; and (4) the real‐time and feedback interface to allow sequence changes during the scan.

### 2.1 General sequence design and generation

**FIGURE 1:**
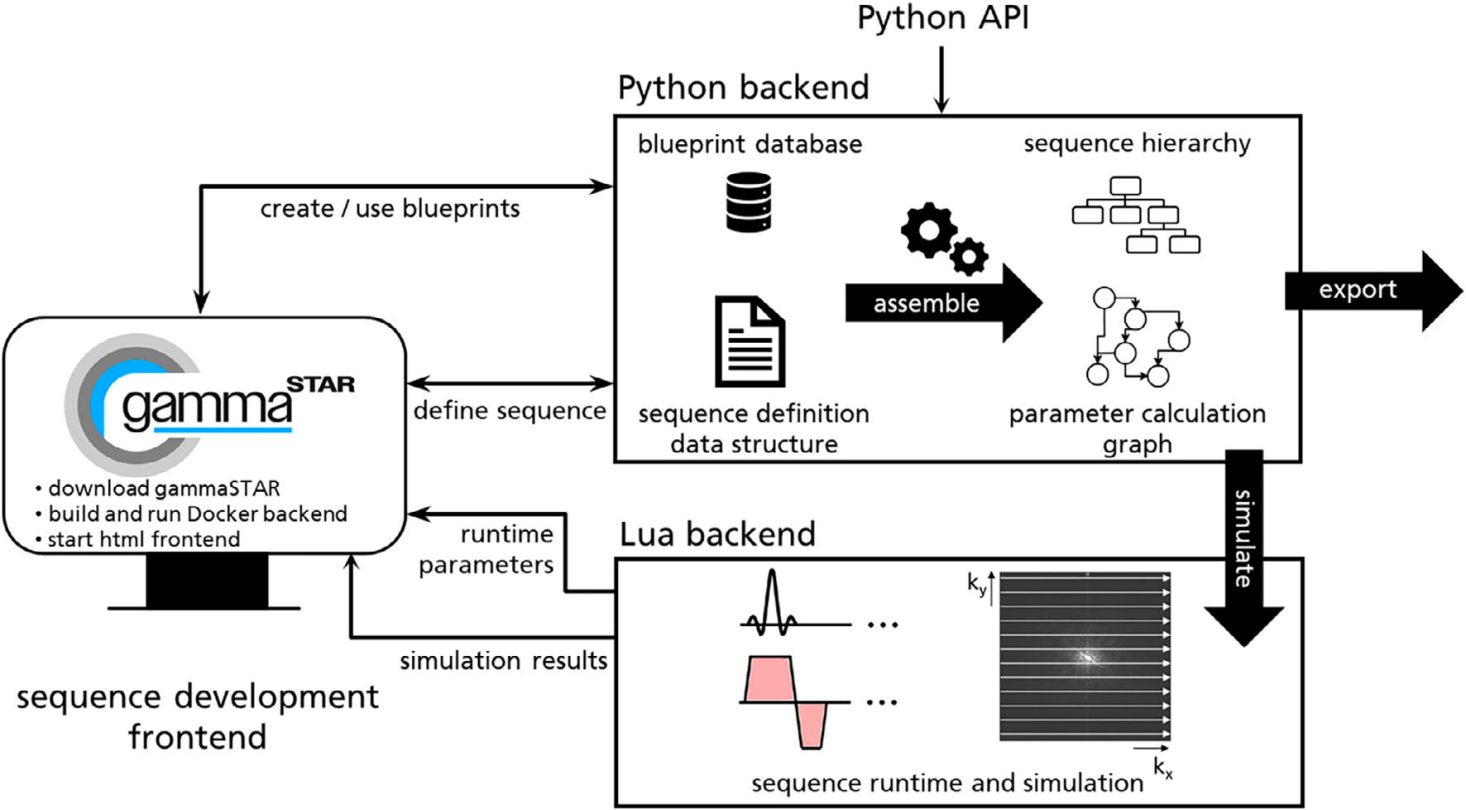
Interaction between web‐based gammaSTAR frontend and Python/Lua backend services. The Python backend holds all information about the available blueprint database and is called for getting existing or creating new sequence modules. It will also be in sync with the gammaSTAR frontend when the sequence developer defines a new sequence from existing blueprints. This sequence definition data structure will be assembled to generate the sequence hierarchy and parameter calculation graph, which can be interpreted by the Lua backend for simulation or sequence execution. These results can be communicated back to the frontend.

The basic concept of generating an MR sequence in gammaSTAR is shown in Figure 1. The user interaction is fully integrated in a web interface communicating with the Python backend code that handles all frontend interactions. The sequence is generated based on an extensible set of definition rules. These resemble reusable modules and physics‐based rules that are called blueprints and defined by the sequence developer using the Lua script language. The blueprints are then combined to an MR sequence that can be generated in an assembly process. Whereas before assembling the sequence, all parameter calculations in the blueprints are formulated by the sequence developer as physics‐based rules without the need of including calculation order (input–output dependencies) or looping, the assembly process generates a well‐defined sequence element hierarchy including loop structures and a parameter graph, which holds a calculation strategy for each involved Lua script by defining how to get all dependencies of this script in the most efficient way. More in‐depth detail on the sequence definition and the assembly process is given in a previous publication.^13^

This information is bundled in the gammaSTAR *.seq.json file to be exported and used at the MR scanner or for offline simulations. Note that this does not represent a static sequence, but still holds the logic to change the sequence based on parameter changes, that is, when changing the sequence protocol at the scanner or applying real‐time feedback. The protocol parameters are accessible via a specific sequence element in the data structure.

### 2.2 gammaSTAR user interface

For creating, modifying, and combining the above‐mentioned blueprints, gammaSTAR offers a Docker version of the backend with the same web‐based frontend.^14^ A manual with step‐by‐step description on how to use gammaSTAR for sequence development is available on the website. The gammaSTAR frontend also offers many helpful tools to support the user in the development process.

### 2.3 Sequence interpretation

**FIGURE 2:**
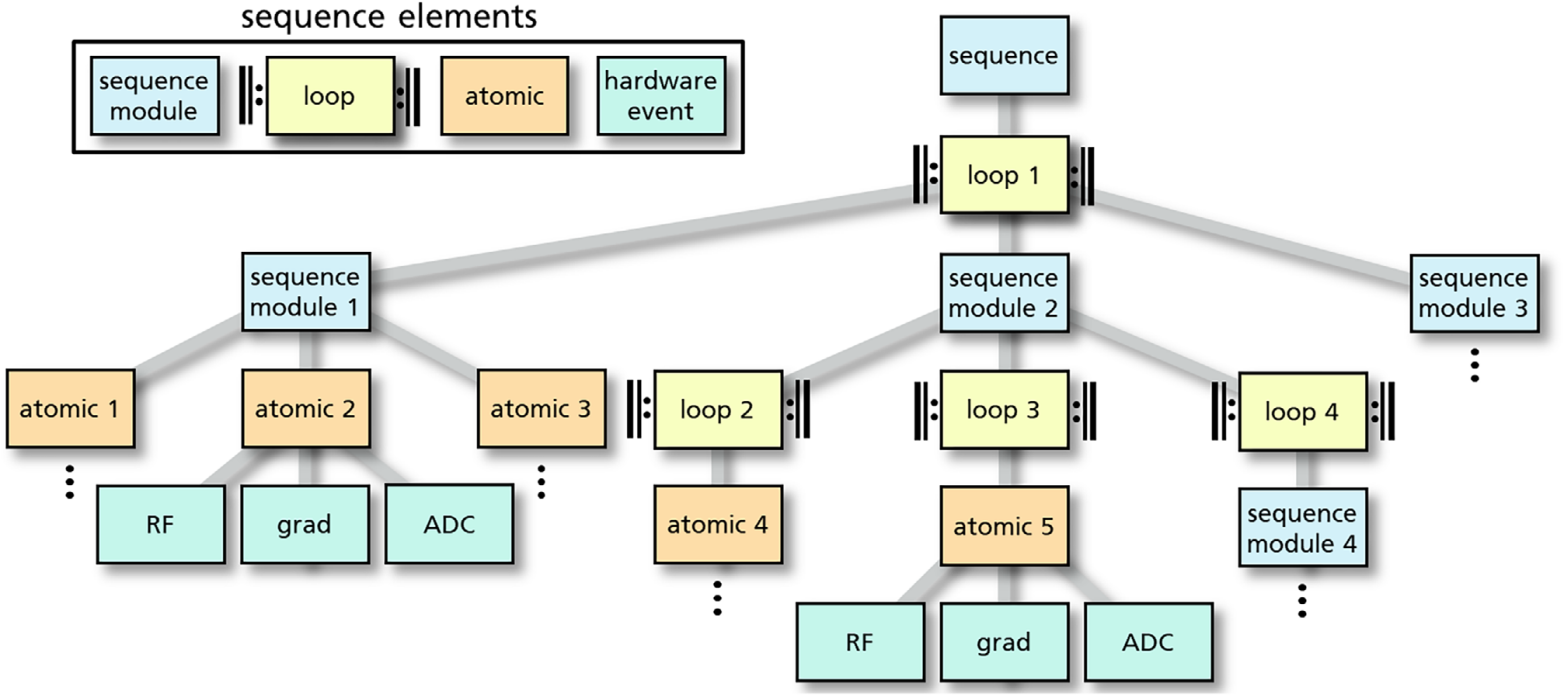
Illustration of the gammaSTAR sequence hierarchy structure, which is exported after developing a sequence in the gammaSTAR frontend alongside the parameter calculation graph. The sequence is defined by different sequence elements, where sequence modules (blue) define the hierarchical structure, loops (yellow) introduce repetitive executions of underlying tree branches and atomics (orange) are flagged sequence modules that are translated into hardware execution blocks of hardware events (green) like RF pulse, gradient, and ADC.

**FIGURE 3:**
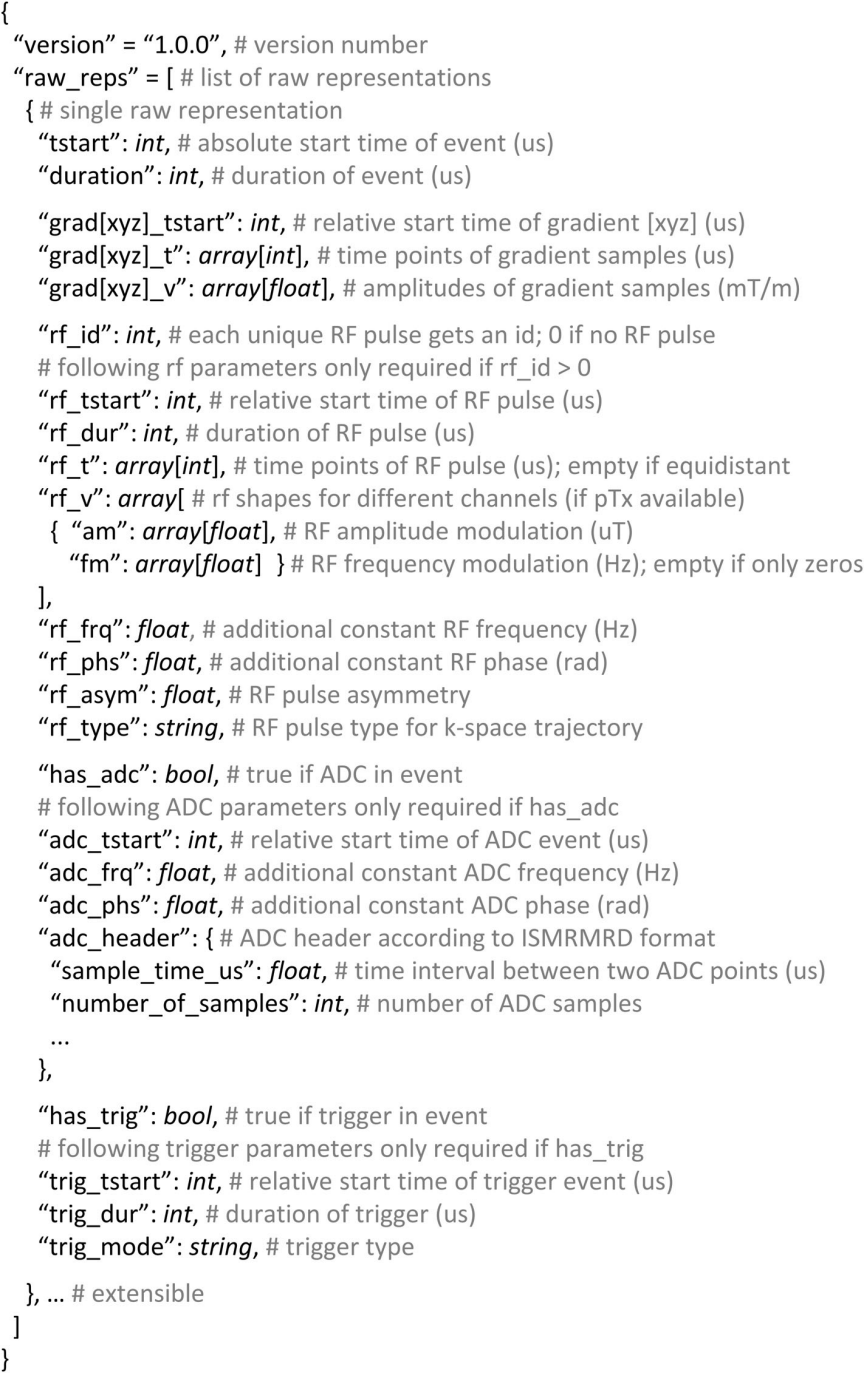
Raw representation of the hardware events. All information (e.g., start times, shapes, and header information) for running the sequence is sent to the driver in this format.

The exported gammaSTAR *.seq.json file that bundles the sequence element hierarchy and parameter graph is input of the gammaSTAR Lua backend, which is used for (1) simulating the sequence offline and sending the results back to the gammaSTAR frontend (Figure 1) and for (2) providing the hardware events for running the sequence at the MR scanner. When interpreting the sequence, the sequence element hierarchy (Figure 2) is traversed. Whenever a loop sequence element is reached, the branch below the loop will get executed for each ascending loop index. If a so‐called atomic sequence element is reached, this sequence element will be translated into single hardware execution blocks and trigger the calculation of involved RF, ADC (+ header) and gradient events by calculating a parameter called raw representation. This calculation is then performed using the exported parameter graph for efficient calculation of all required dependencies. The calculation of a raw representation recursively requests required input values from the parameter graph that are needed for calculating the hardware events. Because of this bottom‐up approach, only calculations that are essential are performed. Calculated parameters are efficiently cached until their inputs change (e.g., because of loops or protocol changes). Every single atomic will generate a raw representation that is a generalized format to define hardware executions in MRI. The format of the gammaSTAR raw representations is given in Figure 3. The ADC header information is used to expose relevant information to the vendors' reconstruction pipeline and/or for using third‐party reconstruction tools.

### 2.4 MR sequence driver

The vendor‐dependent sequence driver is the only interface to the general Lua backend at the MR scanner. This is programmed (1) by including and linking the C‐based Lua interpreter and Lua‐C‐API into C/C++ driver code which allows communication between Lua and C/C++; or (2) using the Lupa library to allow communication between Python and Lua on Python‐based MR systems (e.g., tabletop scanners). The driver also provides access to a dedicated gammaSTAR GUI based on the Fast Light Toolkit (FLTK) library that handles the protocol interactions between the user and gammaSTAR. A feature for checking the validity of the selected protocol is integrated in the GUI. Solve handlers can be integrated to define certain parameter dependencies and adjustments. All required libraries are statically linked to the driver software, avoiding the need of installing files on the MR scanner other than text files (Lua script files and sequence json files).

**FIGURE 4:**
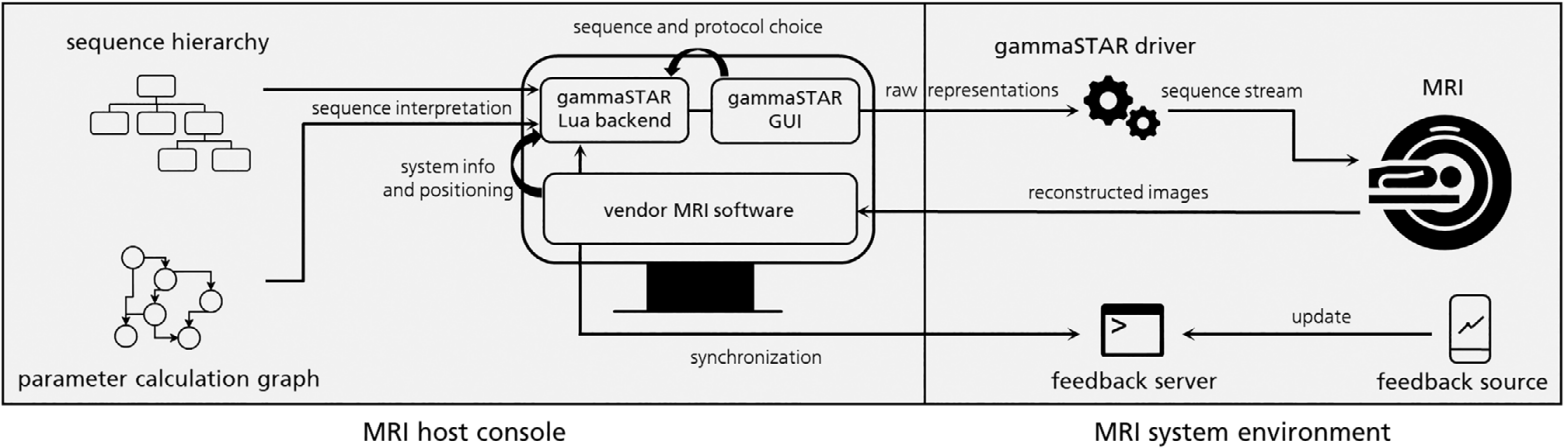
gammaSTAR installation at the MR system. Sequence hierarchy and parameter calculation graph are used as input from the gammaSTAR Lua backend, which is located at the scanner host console. The sequence is initialized using the system parameters and a protocol is chosen using a dedicated gammaSTAR graphical user interface (GUI). Interaction with the vendor MRI software allows for using the familiar positioning process of the imaging plane. When starting the sequence, raw representations are created and sent to the MR system with the gammaSTAR driver installed, which translates the standardized raw representations into scanner‐specific commands. Reconstruction results are sent back to the host console to be used in the image viewer. Additionally, a feedback server is synchronized with the sequence execution and listens to commands from any feedback source.

The interaction of the different components at the MR scanner is depicted in Figure 4. After the initial import of the sequence files, the gammaSTAR GUI provides the Lua backend with the specific protocol. Although sequence‐related parameters are changed via the gammaSTAR GUI at the scanner's host console, the system parameters (e.g., shim, prescan normalization, image scaling) and positioning information are provided by the vendor software. Corresponding vendor parameter fields (e.g., TE, TR, flip angle, resolution) are filled with the values adapted in the gammaSTAR GUI. The generated raw representations are then sent to the gammaSTAR driver, which converts them into scanner‐specific hardware commands. The reconstructed images are available for viewing in the familiar vendor software.

To this date, gammaSTAR sequences can be used with different MR systems. Native gammaSTAR drivers exist for MR devices of Siemens Healthineers (as shown in this manuscript) and Philips Healthcare.^15^, ^16^, ^17^ GE devices can be supported via the intermediate Pulseq format and using TOPPE^6^ as interpreter. gammaSTAR driver code also exists for smaller systems like the OCRA tabletop (MaRCoS^18^) and the ilumr system by Resonint. These developments show the execution of gammaSTAR MR sequences independent of the hardware type, which is made possible by the general format of all software components other than the hardware‐specific driver software. In this manuscript, the driver concept is demonstrated using a single MRI vendor, but different software versions and MRI models.

### 2.5 Real‐time capability and feedback interface

Real‐time capability of MR sequences is given when at any point of the sequence the execution of the current hardware event is in sync with the calculation of the corresponding atomic in the Lua backend. gammaSTAR makes sure to calculate the hardware events in strict chronological order. This means that at any point of the sequence interpretation, all hardware events up to this point are successfully calculated. This also allows the hardware events to be sent to the driver software in a buffered fashion, allowing changes to the sequence between these buffers. It also reduces precalculation time because only the first buffer of hardware events needs to be calculated before starting the scan, which saves loading times and memory and ultimately allows to run clinically relevant and long MR sequences. Without this concept, many of the measurements acquired in this article would not be feasible. It also allows the use of adjacent loop branches that will execute in an interleaved fashion.

**FIGURE 5:**
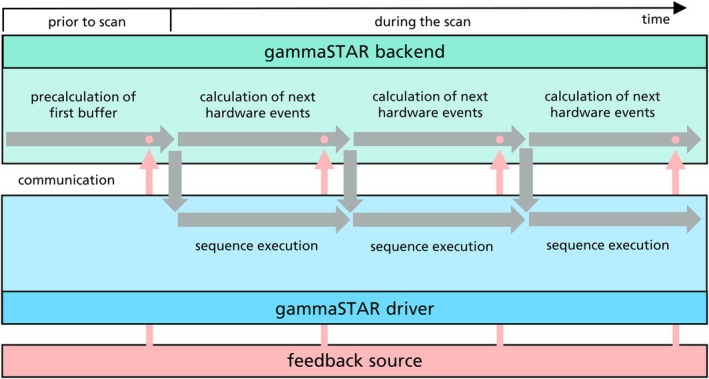
Time‐wise buffering of the sequence execution between gammaSTAR backend (green) and gammaSTAR driver (blue). Only a small part of the sequence is precalculated before the scan and sent from the backend to the scanner sequence execution while subsequent hardware events are being calculated in the backend. The timepoint when the calculated hardware events are sent to the sequence execution can also be used for real‐time synchronization to apply real‐time changes to the sequence from an external feedback source (red).

Although sending the buffer of precalculated hardware events to the MR scanner, the framework can continue traversing the sequence tree to find subsequent hardware events. To reduce computation time, these two processes can be run in separate threads on the scanner. In the case of dynamic sequence changes throughout the scan (e.g., by feedback devices), a synchronization step can be executed for each buffer time (Figure 5), which is typically set to values between 30 and 300 ms. gammaSTAR supports this by running an asynchronous WebSocket server for synchronization between the parameter graph and sequence parameter updates (Figure 4). The user defines beforehand in the sequence which parameters should be available for feedback (e.g., position and orientation of the imaging plane in prospective motion correction applications). When starting the sequence, the Lua backend will initialize the set of parameters and their respective values on the server. At predefined sync points, the Lua backend will get these current parameters, adapt the sequence structure and continue the scan. Without any feedback source, this will not change the sequence execution at all, but as soon as the parameters on the WebSocket server are updated, the sequence will automatically adapt. This WebSocket server can be reached by any external device in the same network, which makes it a very flexible and easy approach.

### 2.6 Pulseq support

gammaSTAR also offers bidirectional Pulseq support, which means that so‐called Pulseq building blocks can be used as gammaSTAR sequence modules in the sequence. These hold a Pulseq format description of a single event, a group of events or a whole sequence as a string. During execution of the gammaSTAR sequence, the Pulseq blocks are translated to the static gammaSTAR representations. They can be integrated as part of the gammaSTAR sequence structure. For exporting Pulseq files, the gammaSTAR sequence is traversed as done when executing the sequence at the scanner and its raw representation is translated to Pulseq block descriptions.

### 2.7 MR sequence library and measurements

The gammaSTAR framework provides many MR imaging and spectroscopy modules and sequences. Different 2D and 3D readout trajectories are available including Cartesian (e.g., FLASH, rapid acquisition with relaxation enhancement [RARE], balanced SSFP [bSSFP], EPI, gradient and spin‐echo [GRASE], periodically rotated overlapping parallel lines with enhanced reconstruction [PROPELLER]) and non‐Cartesian (e.g., radial, spiral, twisted projection imaging [TPI]) readouts for creating different contrasts (e.g., MPRAGE, fluid‐attenuated inversion recovery [FLAIR], pulsed arterial spin labeling [PASL] and Hadamard‐encoded pseudo‐continuous ASL [pCASL] perfusion, diffusion). MR spectroscopy can be performed with STEAM and PRESS sequences. RF pulses with different shapes are implemented, such as sinc, Gaussian, Hanning, frequency offset corrected inversion (FOCI), and Shinnar‐Le Roux (SLR) pulses. Parallel acquisition techniques (GRAPPA and controlled aliasing in parallel imaging results in higher acceleration [CAIPIRINHA]) and 2D‐selective and simultaneous multislice excitation are available for faster image acquisition. Different preparation modules can be combined to suppress MR signals and to achieve different image contrasts (e.g., fat/water suppression, regional saturation, inversion, spin echo [SE], background suppression, Q2TIPS [quantitative imaging of perfusion using a single subtraction, version 2, with thin‐slice TI1 periodic saturation]). The sequence library is continuously extended.

In vivo measurements of the same volunteer were performed on clinical 3 T and 1.5 T MR scanners (MAGNETOM Vida Fit [SW: XA50A] and Avanto Fit [SW: VE11C], Siemens Healthineers) equipped with 20‐channel head coils. Written informed consent before scanning was provided and the study was run under a general protocol for pulse‐sequence development approved by the local ethics committee. The sequence protocol parameters are listed in Table 1.

**TABLE 1:** Sequence protocol parameters used for in vivo measurements.

Protocol	TR/TE/TI [ms]	FA [°] EXC/SE	FOV [mm]	Matrix	PF PHS/SLC	PAT acc. PHS/SLC	Train length PHS/SLC	Av.	Scan time [min:s]	Other
3D bSSFP	5/2.5/−	10/−	256 × 192 × 160	256 × 192 × 160	1/0.75	2/1	−/−	1	1:01	10 linear ramp prescans
3D FLASH	10/5/−	10/−	256 × 192 × 160	256 × 192 × 160	1/0.75	2/1	−/−	1	1:59	
3D MPRAGE	2200/2.9/900	8/−	256 × 192 × 160	256 × 192 × 160	1/0.75	2/1	−/−	1	4:06	
2D RARE	6000/48/−	90/150	256 × 192 × 160	256 × 192 × 32	1/−	2/−	8/−	1	1:24	
2D FLAIR	9000/84/2600	90/150	256 × 192 × 160	256 × 192 × 32	1/−	2/−	12/−	1	2:31	2 slice group concatenations
STEAM	2000/20/−	90/90	25 × 25 × 25	–	−/−	−/−	−/−	96	3:16	2 prescans; 2048 data points; spectral bandwidth of 2000 Hz; mixing time of 10 ms
PRESS	2000/32/−	90/180	25 × 25 × 25	–	−/−	−/−	−/−	96	3:16	2 prescans; 2048 data points; spectral bandwidth of 2000 Hz
2D SE‐EPI diffusion	8000/66.6/−	90/180	240 × 180 × 162	80 × 60 × 54	1/−	2/−	30/−	1	1:15	Fat saturation; bipolar gradients with *b* = [0, 300, 600] s/mm^2^ along 3 axes
3D pCASL GRASE (Hadamard‐8)	4000/17.9/−	90/120	256 × 192 × 160	64 × 48 × 32	1/0.75	2/2	24/12	4	2:15	Fat saturation; Q2TIPS; 1 prescan; background suppression to suppress T_1_ values of 700 and 1400 ms; subbolus duration of 0.4 s; PLD = 0.6 s
3D pCASL GRASE (M0)	5000/17.9/−	90/120	256 × 192 × 160	64 × 48 × 32	1/0.75	2/2	24/12	1	0:19	Fat saturation; Q2TIPS; 1 prescan; TI = [300, 1300, 2300] ms
3D radial UTE	6/[0.1, 1.8]/−	10/−	256 × 256 × 256	192 × 192 × 192	−/−	−/−	−/−	1	2:02	20 000 spokes; 540 prescan spokes for correction; golden angle sampling

*Note*: The vendor's sequence parameters were adjusted accordingly (if possible). No product sequences are available for 3D radial and pCASL with Hadamard encoding.

Abbreviations: Av., averages; bSSFP, balanced SSFP; EXC, excitation; FA, flip angle; FLAIR, fluid‐attenuated inversion recovery; GRASE, gradient and spin‐echo; PAT acc., parallel acquisition technique acceleration; pCASL, pseudo‐continuous arterial spin labeling; PF, partial Fourier; PHS, phase; PLD, post labeling delay; RARE, rapid acquisition with relaxation enhancement; SE, spin echo; SLC, slice.

All Cartesian sequences were reconstructed using the scanner's standard reconstruction pipeline. For image reconstruction of non‐Cartesian sequences gridding with a Kaiser‐Bessel kernel^19^ (2× oversampling, window width = 4.0) followed by a complex fast Fourier transformation and deconvolution was integrated into the vendor's reconstruction pipeline. Evaluation of the single‐voxel spectroscopy (SVS) data were performed offline in Python to ensure the same evaluation algorithm for gammaSTAR and the product sequence.

A whole‐brain scan was performed with sequences exported from gammaSTAR and compared with the vendor's product sequences at two different magnetic field strengths. Parameters were kept as consistent as possible because no information about the manufacturer's sequence schemes is available. The scan times between the gammaSTAR and the product sequences can slightly differ because of differing implementations of some modules (e.g., parallel acquisition technique reference scan). Small deviations in image quality and contrast can occur because of different slice profiles and other parameters. The following sequences, with protocol parameters listed in Table 1, were measured for comparison: 3D bSSFP, 3D FLASH, 3D MPRAGE, 2D RARE, 2D FLAIR, STEAM, and PRESS.

1H MR spectroscopy data were measured with the voxel positioned in the medial parietal lobe. The gammaSTAR's STEAM^20^, ^21^ and PRESS^22^ sequences consisted of variable power RF pulses with optimized relaxation delays (VAPOR) water suppression^23^ and interleaved outer‐volume suppression blocks. A water reference scan with one average was acquired for coil combination. The spectra were obtained by simple Fourier transformation after removal of the first acquisition points without any filtering or other postprocessing steps.

The ADC map was calculated from a 2D SE‐EPI gammaSTAR acquisition with bipolar diffusion gradients applied along three orthogonal axes with *b*‐values of [0, 300, 600] s/mm^2^. The geometric mean of these images was calculated to generate isotropic diffusion‐weighted images for the different *b*‐values. A least squares curve fitting algorithm was applied pixelwise to calculate the ADC map from the exponential signal decay curve *S*(*b*) = *S*_0_ exp(−*bD*), where *S*_0_ the signal of the *b* = 0 scan and *D* = ADC.

A pCASL sequence with GRASE readout^24^ was used to achieve perfusion‐weighted images (protocol parameters listed in Table 1). The parallel acquisition technique CAIPIRINHA^25^ was applied to obtain single‐shot acquisitions with an acceleration factor of two along phase and slice direction. Two FOCI pulses suppressed T_1_ values of 700 ms and 1400 ms for background suppression. A Walsh‐ordered Hadamard‐8 labeling encoding scheme and a M0 image without labeling and background suppression was acquired for perfusion quantification. The M0 scan was repeated with phase reversed direction for distortion correction using TOPUP.^26^ The Bayesian inference method of the Oxford Centre for Functional MRI of the Brain's software library^27^ was used for perfusion analysis with following value assumptions at 3 T: T_1_/T_2_ values of 1.331/0.085 s and 1.664/0.165 s for tissue and blood, respectively.^28^

The 3D radial UTE sequence with golden angle sampling^29^ shows the image quality of a non‐Cartesian sequence. A readout gradient delay of 10 us to the ADC was applied to allow for a k‐space trajectory correction by means of 180 different angle prescans in the xy, xz, and yz planes with an additional small dephaser gradient.^30^ The gradient delays were calculated for all three axes and used to determine the actual k‐space trajectory of each spoke. Two echoes were acquired to be able to visualize short T_2_* components by image subtraction.

For demonstrating the real‐time feedback feature of gammaSTAR, a SE‐EPI sequence with TE/TR values of 100/3000 ms was acquired without head restraint for 5 min to obtain 100 measurements with 12 slices of 4 mm isotropic voxel size. Measurements were performed with and without prospective motion correction. The raw data were transferred to an external computer immediately after acquisition where the reconstruction and motion detection were performed on‐the‐fly using a combination of Gadgetron^31^ and ITK using a volume‐to‐volume least‐squares based image registration to the first acquired volume, as demonstrated in the conventional prospective acquisition correction^32^ approach. The new slice position and orientation was sent to the gammaSTAR WebSocket server such that the ongoing measurement was corrected. Mean voxel displacement^33^ values were calculated for both prospectively corrected and uncorrected acquisitions.

## 3 RESULTS

**FIGURE 6:**
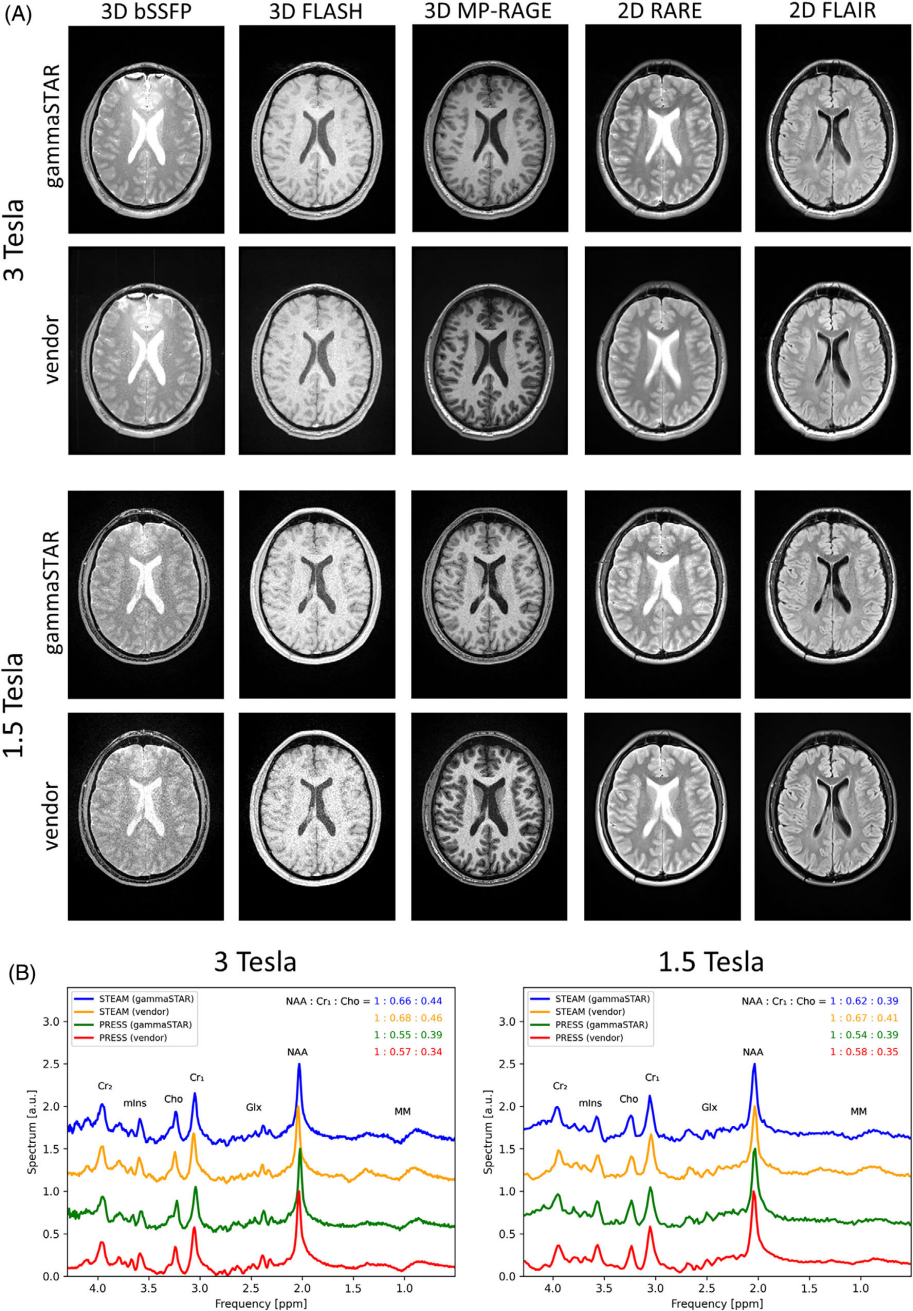
(A) Comparison of different gammaSTAR sequences with the corresponding vendor‐provided sequences at two different magnetic field strengths. (B) Spectrum of single‐voxel spectroscopy acquired with gammaSTAR and vendor implementations of the STEAM and PRESS sequences. The voxel was placed into the medial parietal lobe. Ratios of peak amplitudes are shown in the upper right corner. Cho, choline; Cr, creatine; Glx, glutamate and glutamine; mIns, myo‐inositol; MM, macromolecules; NAA, N‐acetylaspartate.

In Figure 6, the whole‐brain gammaSTAR acquisitions are compared with the corresponding sequences by the vendor at magnetic field strengths of 3 and 1.5 T (see Table 1 for protocol parameters). Figure 6A shows image results exemplarily for one slice for 3D bSSFP, 3D FLASH, 3D MPRAGE, 2D RARE, and 2D FLAIR. The image qualities and contrasts are quite similar except for small deviations in the 3D MPRAGE images between gammaSTAR and vendor scans. In Figure 6B, the comparison of the SVS sequences also show similar spectra for the STEAM and PRESS scans at both magnetic field strengths. Water is sufficiently suppressed and all main metabolites are clearly visible. Ratios of the peak amplitudes are given in the upper right corner.

**FIGURE 7:**
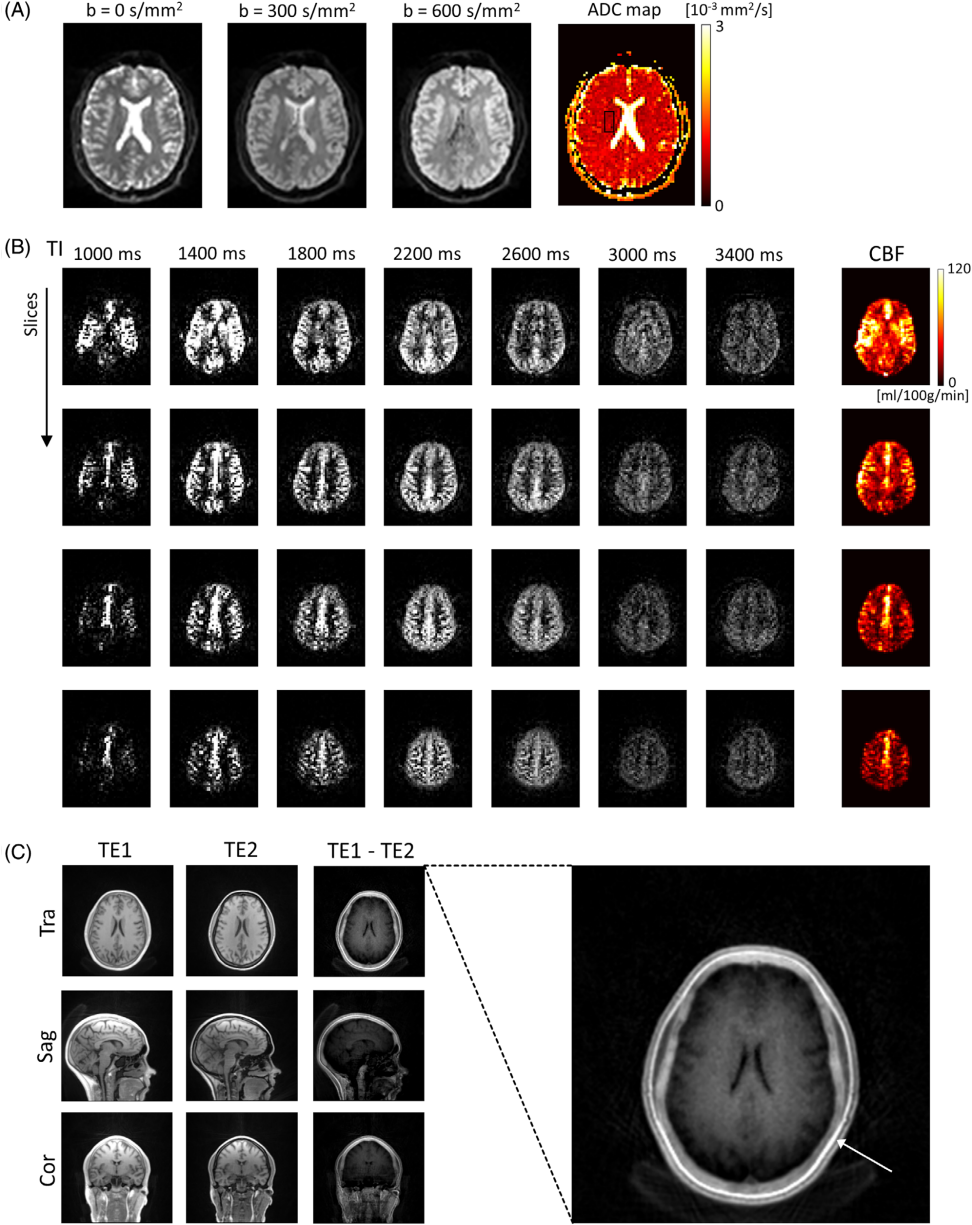
(A) Trace‐weighted images and ADC map obtained by the diffusion‐weighted 2D spin echo EPI gammaSTAR sequence acquired with *b*‐values of [0, 300, 600] s/mm^2^. (B) Perfusion‐weighted images (exemplarily shown for four slices) acquired with the gammaSTAR 3D pseudo‐continuous arterial spin labeling gradient and spin‐echo (pCASL GRASE) sequence with Hadamard‐8 encoding. All resulting images of the seven inflow times are shown with the calculated cerebral blood flow (CBF) map. (C) 3D radial UTE images of both echoes (TE1 and TE2) in transverse, sagittal and coronal view. The difference image (TE1‐TE2) shows short T_2_* components like the skull bone (arrow).

In Figure 7, the physiological imaging results acquired with the gammaSTAR sequences at 3 T are summarized. Figure 7A shows the trace‐weighted diffusion images and the resulting ADC map obtained by the 2D SE‐EPI sequence. The region of interest‐based analysis of the ADC values in white matter resulted in (0.80 ± 0.14)10^−3^ mm^2^/s. The perfusion‐weighted images acquired with the Hadamard‐encoded pCASL sequence with inflow times ranging from 1000 to 3400 ms are demonstrated in Figure 7B. Four slices are exemplarily shown with the corresponding cerebral blood flow (CBF) values calculated from the perfusion‐weighted images and the M0 scans. The blood inflow is clearly visible and no severe image artifacts can be observed. The results of the 3D radial UTE scan are shown in Figure 7C. Both echo time images TE1 and TE2 show minor undersampling artifacts. They are strongly reduced in the difference image TE1‐TE2, where the skull bone can be delineated very well (see arrow).

**FIGURE 8:**
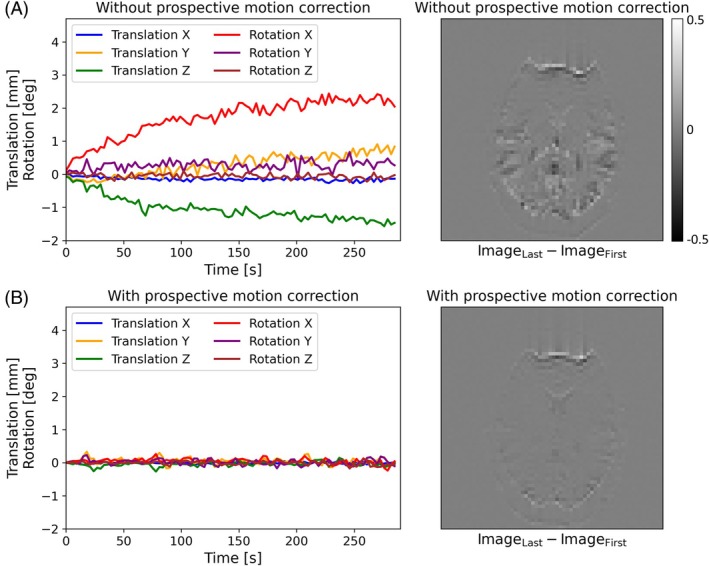
Translational and rotational motion shown for the case (A) without and (B) with prospective motion correction. On the right, the difference images of the last and first measurement are shown for both cases.

Figure 8 shows the residual rigid motion parameters (Figure 8A) without and (Figure 8B) with prospective motion correction. The subject showed a severe drifting motion pattern, which was not apparent when scanning with motion correction turned on. The difference image of the last and first image shows much fewer movement artifacts in the case of motion correction, which is confirmed by mean voxel displacement values of 1.63 and 0.37 mm for the uncorrected and corrected acquisition, respectively. Low difference signals can still be observed in the front area of the head in case of prospective correction.

Because of the buffered real‐time sequence execution all sequences had a starting/precalculation time of below 1 s, excluding shimming and system adjustments.

## 4 DISCUSSION

This article demonstrates a mature framework for developing and executing MR sequences. This section will compare the features to existing frameworks and discuss the measurement results. It will also bring the work in context to the current MRI market and open science movement.

### 4.1 Comparison with other frameworks

A trade‐off between ease of use and flexibility usually exists for creating development frameworks. Although Pulseq^5^, ^6^, ^11^, ^12^ offers a user‐friendly format and supporting tools, its lack of features like dynamic protocol changes and real‐time feedback adaptations does not support certain applications. The sequence export is valid for one specific parameter configuration and also needs to be recalculated for MR scanners with higher hardware limitations. The use of inline vendor reconstruction pipelines is limited. In general, the use case of Pulseq sequences is rapid prototyping and not providing product‐like MR sequences. From a sequence development point of view it is clear that as it mainly describes a file format, Pulseq allows more freedom and interoperability between different programming platforms (e.g. Python, MATLAB). For prototyping static MR sequences, Pulseq is more practical with less learning effort. In terms of flexibility, changing a Pulseq sequence requires a new export and transfer of the file to the MR system. gammaSTAR uses a different approach where the sequence logic is not only inside the program code that is producing the sequence export, but saved with the sequence format itself, offering the possibility to allow (1) on‐the‐fly changes of the sequence protocol at the MRI system; and (2) changes of the sequence during the scan. Execution of Pulseq sequences on GE systems is mainly achieved by a conversion of the Pulseq file to the so called TOPPE^6^ platform. TOPPE was developed as distinct sequence platform, but often used as a GE interpreter of Pulseq sequences and therefore shares the same limitations and characteristics as explained above.

JEMRIS^7^ is an open‐source MRI simulation framework, which provides a GUI in MATLAB for sequence design, coil layout, and MRI simulations. A sequence export in the Pulseq format is available and therefore offers same functionality as described in the previous section.

The Object‐Oriented Development Interface for NMR (ODIN)^8^ provides a platform‐independent data processing and sequence design with several predefined C++ class objects. Two interpreters are currently available for running sequences at Siemens and Bruker systems. Although standard sequences with short scan times can be traversed at runtime on Siemens machines, it is reported that a text file with sequential hardware instructions is sent to Bruker systems. Similar to ODIN, SequenceTree^9^ also consists of C++ class modules that are arranged in a hierarchical structure to create sequences. Only a Siemens interpreter is available so far, which is not connected to the image reconstruction pipeline at the scanner. Because sequences are based on C++ for ODIN and SequenceTree, a straightforward interface implementation to scanners with programming languages other than C++ is not feasible.

Full real‐time customization of sequences is not possible for all of the above‐mentioned programming platforms, or at least the capability is not reported to exist. The main focus of gammaSTAR is to create a suitable sequence format maintaining the manufacturers' features without sacrificing too much user‐friendliness. This is achieved by still keeping sequence logic and dependencies in the sequence export. A previously described parameter graph^13^ and the buffering of the sequence events allow sequence streaming in real‐time, even of very challenging sequences such as pCASL. In contrast to the platforms mentioned above, gammaSTAR allows for sequence adaptations during the scan via the driver/Lua backend or a WebSocket.

### 4.2 Real‐time capability

The gammaSTAR sequences are run in a real‐time format to minimize memory consumption and computation times. It allows running clinically relevant protocols without wait times despite long acquisition times as shown by the imaging results in this manuscript. This is established by traversing the sequence tree in chronological order and buffering only small parts of the sequence. This execution window is typically set to 30 to 300 ms. When synchronization at certain positions is required (e.g., correction of the imaging plane position as response to subject motion right before slice excitation), additional sync points are added automatically at these points of interest. For typical applications that require feedback to the sequence, such as prospective motion correction, electrocardiogram triggering, or automatic slice steering, the current timing is adequate, as demonstrated by the results in this manuscript. However, the potential for achieving true event‐based synchronization between the gammaSTAR runtime component and hardware execution is an area that warrants further investigation.

Exploring this capability could eventually enable direct control over spectrometers in open‐source and low‐field MR systems, enhancing the flexibility and applicability of gammaSTAR. At present, gammaSTAR maintains synchronization with hardware execution within the time range of the selected execution windows, as discussed earlier.

### 4.3 Measurements

Measurements with different standard MR pulse sequences were performed at two different magnetic field strengths and compared to the product sequences of the vendor (Figure 6A). Image contrasts and qualities are quite similar, but the contrast of the 3D MPRAGE acquisition slightly differ, which can be explained by minor deviations in sequence timing and different definitions of pulse shapes. Since the pulse scheme of the vendor is not known, protocol parameters and pulse shapes cannot be adjusted and compared correctly. For instance, there is no standard definition of a conventional sinc RF pulse and each vendor uses its individual time‐bandwidth product parameter. This difference in the slice profile can also be observed in the cerebrospinal fluid region of the 2D RARE acquisition.

The quantitative evaluation of the diffusion‐weighted MRI also shows good agreement of ADC values (0.80 ± 0.14)10^−3^ mm^2^/s in white matter with literature values of (0.70 ± 0.03)10^−3^ mm^2^/s with range (0.62–0.79)10^−3^ mm^2^/s in different subjects.^34^ The spectra of the SVS sequences are very similar to those acquired with the vendor sequences. The different peak amplitudes of PRESS and STEAM arise from the fact that they have deviating echo times and because of their different sensitivity to J coupling.

The results of the pCASL GRASE, and 3D radial sequences (Figure 7) demonstrate the application of more challenging sequences that are not available in the standard MR software package by the vendor. As mentioned above in the section on real‐time capability, the gammaSTAR real‐time feedback with its buffering technique allows running more complex sequences. CBF maps of the whole brain with sufficiently high quality can be acquired within approximately 3 min considering also both M0 images. Common artifacts of non‐Cartesian MR sequences could be reduced by a k‐space trajectory correction method, which is necessary to clearly delineate the skull bone in the difference image of both echoes.

The prospective motion correction application (Figure 8) further demonstrates the need of the real‐time feedback capability, which is not available in other development frameworks.^5^, ^6^, ^7^, ^8^, ^9^ This is possible in gammaSTAR and applicable to any other application where real‐time feedback is required such as correction for frequency shifts. The motion artifacts could be reduced tremendously, whereas the susceptibility artifacts in the front area of the head cannot completely corrected for.

### 4.4 Safety

Safety is an important topic when using third‐party sequence development software. gammaSTAR supports the user during sequence implementation with useful information regarding peripheral nerve stimulation (PNS) and RF energy. However, in the driver software, the vendor processes to secure patient safety also need to be followed. All gammaSTAR drivers follow the recommendations and regulations on how to communicate RF and gradient events for calculating specific absorption rate and PNS levels to the system such that the vendor models for energy absorption and nerve stimulation can be used as done in a vendor sequence. This is similar to sequences that are implemented via the manufacturer‐specific development software.

### 4.5 Open‐source and availability

The gammaSTAR framework consists of several software components. The driver component is based on proprietary vendor code and therefore can only be shared with agreement by the vendor and through the vendors' sharing platforms. gammaSTAR provides an open access to frontend and backend functionality that is available online.^14^ An extensive open‐source sequence library already exists, which shall be extended in a community effort to support the open science initiative.

Besides the vendor‐specific driver, gammaSTAR can be used in a hardware‐agnostic environment supporting standardized raw data format (ISMRMRD^35^) and open‐source image reconstruction (Gadgetron^31^). This would support comparability between different imaging platforms. Since this manuscript compares different systems from the same vendor, the images presented are reconstructed using the vendor's proprietary platforms. The evaluation of reconstruction results using the vendor's proprietary platform against open‐source frameworks like Gadgetron in measurements across different hardware and vendors remains a topic of future work, some of which have already been presented.^16^

## 5 CONCLUSION

The framework gammaSTAR for MR sequence development with real‐time feedback is presented. In vivo measurements show that image qualities and contrasts of the different MR sequences are comparable to product sequences. gammaSTAR allows for sequence adaptations during the scan via its real‐time interfaces, which could be demonstrated by the prospective motion correction results.

## Data Availability

The source code of the sequence database used in the frontend at https://gamma‐star.mevis.fraunhofer.de can be found at https://github.com/FraunhoferMEVIS/gammaSTAR.
